# Supplementary material for: Opioid Free Anesthesia in Thoracic Surgery: A Systematic Review and Meta Analysis
Source: J Clin Med. 2022 Nov 25;11(23):6955. doi: 10.3390/jcm11236955 (PMC9740730; doi:10.3390/jcm11236955)

Supplemental Material to

Opioid free anesthesia in thoracic surgery: a systematic review and meta-analysis.

Filippo D'Amico, Gaia Barucco, Margherita Licheri, Gabriele Valsecchi, Luisa Zaraca, Marta Mucchetti, Alberto Zangrillo, Fabrizio Monaco

Supplementary material Table S1. Postoperative analgesia.

Supplementary material Figure S1. Sensitivity analysis for complications. Forest plot of risk ratio to develop complications comparing opioid-free anesthesia and opioid-based anesthesia.

Supplementary material Figure S2. Sensitivity analysis for morphine equivalent consumption at 48 hours . Forest plot of mean difference of morphine equivalent consumption at 48 hours comparing opioid-free anesthesia and opioid-based anesthesia.

Supplementary material Figure S3. Sensitivity analysis for length of stay in patients undergoing minimally invasive surgery. Forest plot of mean difference of length of stay comparing opioid-free anesthesia and opioid-based anesthesia.

Supplementary material Figure S4. Sensitivity analysis for pain score at 24 hours. Forest plot of mean difference of pain at 24 hours comparing opioid-free anesthesia and opioid-based anesthesia.

Supplementary material Figure S5. Sensitivity analysis to detect the source of heterogeneity in morphine equivalent consumption at 48 hours. Forest plot of mean difference of morphine equivalent consumption at 48 hours comparing opioid-free anesthesia and opioid-based anesthesia

Table S1

Study	Postoperative analgesia
**Bello, 2019 [16]**	• PCEA for 48 hours using ropivacaine 0.2% with sufentanil 0.25 $mg.mL^{-1}$<br>• Paracetamol (1 g) every 6 hours during the first 48 hours<br>• Rescue analgesia (nefopam, tramadol, ketoprofene or morphine PCA.
**Clark, 2022 [20]**	• Gabapentin 300 mg every 8 hours<br>• Acetaminophen 1000 mg every 8 hours<br>• Methocarbamol 500 mg of every 6 hours<br>• Rescue dose: 30 mg ketorolac every 6 hours
**Devine, 2020 [19]**	• Extra- pleural continuous analgesia: levobupivacaine 0.25%<br>• Morphine PCA for the first postoperative 24 hours<br>• Paracetamol p.o.<br>• Ibuprofen
**An, 2021 [21]**	N/A
**Larue, 2022 [18]**	• Morphine<br>• Tramadol
**Selim 2021 [17]**	• NRS>3 paracetamol (1 g) every 6 hours, nefopam (20mg) every 6 hours, ketoprofen (100 mg) every 12<br>• NRS 6-10 morphine sulfate (10 mg) orally, every 6 hours.<br>• Rescu analgesia: morphine PCA

PCEA: Patient Control Epidural Analgesia; PCA: Patient Control Analgesia; N/A: Not Applicabile; NRS: Numerical Rating Scale

Figure S1

Study or Subgroup	OFA Events	OFA Total	OBA Events	OBA Total	Weight	Risk Ratio M-H, Fixed, 95% CI
Bello 2019	10	25	21	50	0.0%	0.95 [0.53, 1.70]
Clark 2022	64	102	178	211	98.5%	0.74 [0.63, 0.87]
Selim 2021	0	48	1	33	1.5%	0.23 [0.01, 5.51]
**Total (95% CI)**		**150**		**244**	**100.0%**	**0.74 [0.63, 0.86]**
Total events	64		179			

Heterogeneity: Chi² = 0.53, df = 1 (P = 0.47); I² = 0%
Test for overall effect: Z = 3.73 (P = 0.0002)

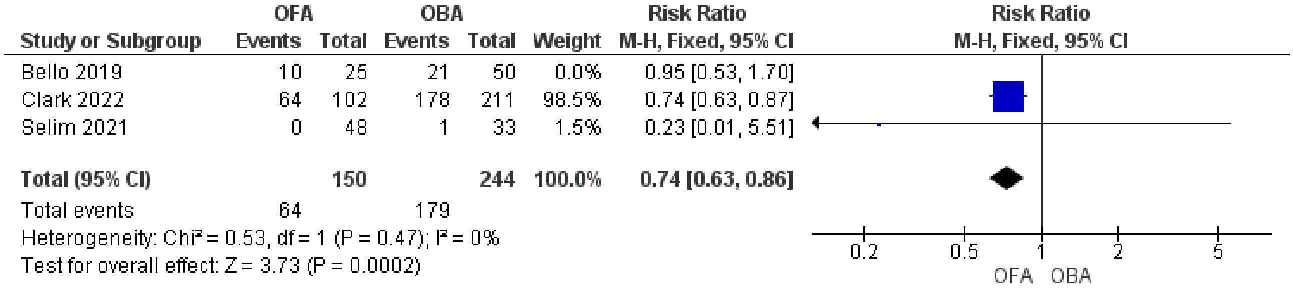

Figure S2

Study or Subgroup	OFA Mean	OFA SD	OFA Total	OBA Mean	OBA SD	OBA Total	Weight	Mean Difference IV, Fixed, 95% CI
Bello 2019	0	0.2	25	8	4.3	50	0.0%	-8.00 [-9.19, -6.81]
Larue 2022	78	60	52	85	49.1	99	7.1%	-7.00 [-25.96, 11.96]
Selim 2021	29	14	48	55	10	33	92.9%	-26.00 [-31.23, -20.77]
**Total (95% CI)**			**100**			**132**	**100.0%**	**-24.66 [-29.70, -19.62]**

Heterogeneity: Chi² = 3.59, df = 1 (P = 0.06); I² = 72%
Test for overall effect: Z = 9.59 (P < 0.00001)

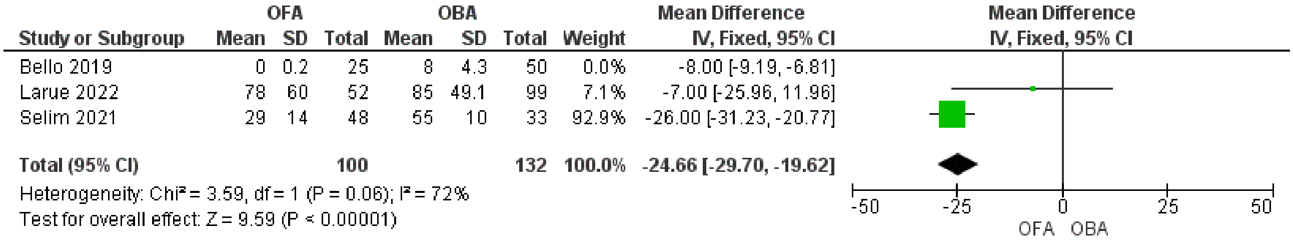

Figure S3

Study or Subgroup	OFA Mean	OFA SD	OFA Total	OBA Mean	OBA SD	OBA Total	Weight	Mean Difference IV, Fixed, 95% CI
Clark 2022	4.2	3.7	102	5.2	5.5	211	53.1%	-1.00 [-2.03, 0.03]
Devine 2020	3	0.6	83	4	1.3	104	0.0%	-1.00 [-1.28, -0.72]
Larue 2022	5.6	3.2	52	5.8	3.4	99	46.9%	-0.20 [-1.30, 0.90]
**Total (95% CI)**			**154**			**310**	**100.0%**	**-0.62 [-1.38, 0.13]**

Heterogeneity: Chi² = 1.08, df = 1 (P = 0.30); I² = 8%
Test for overall effect: Z = 1.63 (P = 0.10)

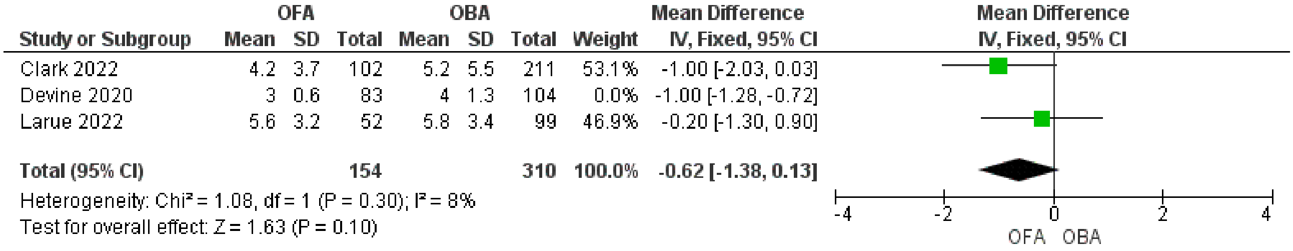

Figure S4

Study or Subgroup	OFA Mean	OFA SD	OFA Total	OBA Mean	OBA SD	OBA Total	Weight	Mean Difference IV, Random, 95% CI
Bello 2019	1	0.5	25	3.8	0.8	50	49.9%	-2.80 [-3.10, -2.50]
Devine 2020	0.1	0.1	83	0.1	0.1	113	50.1%	0.00 [-0.03, 0.03]
Selim 2021	0.2	0.6	48	2.5	1.2	33	0.0%	-2.30 [-2.74, -1.86]
**Total (95% CI)**			**108**			**163**	**100.0%**	**-1.40 [-4.14, 1.35]**

Heterogeneity: Tau² = 3.91; Chi² = 340.74, df = 1 (P < 0.00001); I² = 100%
Test for overall effect: Z = 1.00 (P = 0.32)

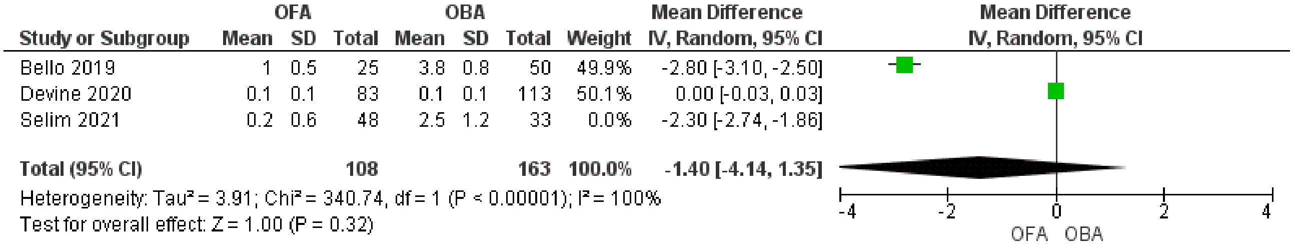

Figure S5

Study or Subgroup	OFA Mean	OFA SD	OFA Total	OBA Mean	OBA SD	OBA Total	Weight	Mean Difference IV, Fixed, 95% CI
Bello 2019	0	0.2	25	8	4.3	50	99.6%	-8.00 [-9.19, -6.81]
Larue 2022	78	60	52	85	49.1	99	0.4%	-7.00 [-25.96, 11.96]
Selim 2021	29	14	48	55	10	33	0.0%	-26.00 [-31.23, -20.77]
**Total (95% CI)**			**77**			**149**	**100.0%**	**-8.00 [-9.19, -6.80]**

Heterogeneity: Chi² = 0.01, df = 1 (P = 0.92); I² = 0%
Test for overall effect: Z = 13.15 (P < 0.00001)

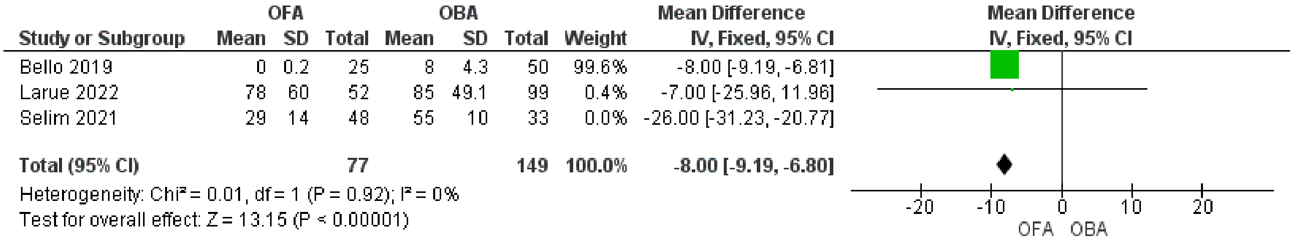

Supplement: Supplementary file 1 [file jcm-11-06955-s001.zip › jcm-2016359-supplementary.pdf]
